# A NWB-based dataset and processing pipeline of human single-neuron activity during a declarative memory task

**DOI:** 10.1038/s41597-020-0415-9

**Published:** 2020-03-04

**Authors:** N. Chandravadia, D. Liang, A. G. P. Schjetnan, A. Carlson, M. Faraut, J. M. Chung, C. M. Reed, B. Dichter, U. Maoz, S. K. Kalia, T. A. Valiante, A. N. Mamelak, U. Rutishauser

**Affiliations:** 1 Department of Neurosurgery, Cedars-Sinai Medical Center, Los Angeles, CA USA; 2 Institute for Interdisciplinary Brain and Behavioral Sciences, Crean College of Health and Behavioral Sciences, Schmid College of Science and Technology, Chapman University, Orange, CA USA; 3 Krembil Brain Institute, Toronto Western Hospital, Toronto, Canada; 4 Department of Neurology, Cedars-Sinai Medical Center, Los Angeles, CA USA; 5 Biological Systems & Engineering Division, Lawrence Berkeley National Laboratory, Berkeley, CA USA; 6 Department of Neurosurgery, Stanford University, Stanford, CA USA; 7 Division of Biology and Biological Engineering, California Institute of Technology, Pasadena, CA USA; 8 Division of Neurosurgery, Department of Surgery, University of Toronto, Toronto, Canada; 9 Computational and Neural Systems Program, California Institute of Technology, Pasadena, CA USA; 10 Center for Neural Science and Medicine, Department of Biomedical Science, Cedars-Sinai Medical Center, Los Angeles, CA USA

**Keywords:** Long-term memory, Software, Data publication and archiving

## Abstract

A challenge for data sharing in systems neuroscience is the multitude of different data formats used. Neurodata Without Borders: Neurophysiology 2.0 (NWB:N) has emerged as a standardized data format for the storage of cellular-level data together with meta-data, stimulus information, and behavior. A key next step to facilitate NWB:N adoption is to provide easy to use processing pipelines to import/export data from/to NWB:N. Here, we present a NWB-formatted dataset of 1863 single neurons recorded from the medial temporal lobes of 59 human subjects undergoing intracranial monitoring while they performed a recognition memory task. We provide code to analyze and export/import stimuli, behavior, and electrophysiological recordings to/from NWB in both MATLAB and Python. The data files are NWB:N compliant, which affords interoperability between programming languages and operating systems. This combined data and code release is a case study for how to utilize NWB:N for human single-neuron recordings and enables easy re-use of this hard-to-obtain data for both teaching and research on the mechanisms of human memory.

## Abstract (summary)

**Table:** 

Measurement(s)	medial temporal lobe • memory • amygdala • hippocampus
Technology Type(s)	single-unit recording
Factor Type(s)	confidence • remembered/forgotten • old/new • visual category • stimulus onset (visual)
Sample Characteristic - Organism	Homo sapiens

Machine-accessible metadata file describing the reported data: 10.6084/m9.figshare.11835801

## Background & Summary

*In-vivo* experiments in awake, behaving animals produce a large and complex mixture of different types of data, which is typically stored in a heterogeneous set of files formatted in a variety of equipment-or laboratory specific data formats. As a result, it is a challenge to share such data for re-use by others to, for example, perform meta-analysis across datasets. To enable the wide-reuse and sharing of systems neuroscience data, it is instrumental to utilize a standardized data format capable of storing all elements associated with an experiment. Key requirements for such a standard^1–3^ include the ability to store large-scale complex data and meta data, language-and platform independent accessibility, extensibility for custom use cases, and easy usability for neuroscientists.

While various file formats have been introduced to address the requirements noted^4–9^, a universally accepted standard has yet to emerge for the storage of cellular-level data. A comprehensive, standardized data format satisfying these requirements that is suitable for the storage of cellular-level imaging and electrophysiology data has recently emerged: the Neurodata Without Borders: Neurophysiology 2.0 format (NWB:N)^1–3^. NWB:N is designed to store both raw and processed data and associated metadata for diverse types of imaging and electrophysiology data. NWB:N provides APIs for both Python and MATLAB to store, query, and retrieve data in a platform and programming language independent manner. NWB:N utilizes HDF5 (Hierarchical Data Format) (see https://www.hdfgroup.org/solutions/hdf5/) as a storage backend, which is well-suited to store large amounts of data and which is supported by many programming languages (including Python, MATLAB, and C++), assuring accessibility and interoperability of NWB:N (see https://neurodatawithoutborders.github.io/storage_hdf). Within NWB:N, data is organized according to the following primitives: Groups (which are similar to a folder), Datasets (n-Dimensional Data tables), Attributes (the meta-data), and Links (References to Datasets). The NWB standard makes use of these primitives to organize all data associated with an experiment (see https://neurodatawithoutborders.github.io/schemalanguage).

Here, we describe how we exported a complex, large dataset of single neuron recordings from the human medial temporal lobe and behavior to the NWB:N format^10,11^ and show how to import and use the NWB:N-formatted data to perform single-neuron analysis. The goal of this release is four-fold: (i) to demonstrate the feasibility of using NWB:N for human single-neuron studies, (ii) to demonstrate that the resulting NWB:N files are fully interoperable between programming languages, (iii) to provide MATLAB and Python code templates that can be used by others, and (iv) to release a large human single-neuron dataset as NWB:N (as part of a new NIH BRAIN initiative consortium, we added 17 subjects and 288 neurons, including from a new study site, relative to our previously released dataset, which used a proprietary format^10^). All NWB operations were executed using the standard NWB:N Python (PyNWB, version 1.1.0) and MATLAB (MatNWB, version 0.2.1) APIs, which we utilized to both export our data as well as to re-import it for analysis.

The data described here was recorded extracellularly from individual neurons in the human medial temporal lobe (MTL) in patients with intractable epilepsy^12,13^. Patients were implanted with hybrid depth electrodes with embedded microwires for the purpose of identifying their seizure focus^12,14^. We recorded the activity of single neurons during the administration of a new/old recognition memory task that we and others have used extensively to investigate the neural basis of declarative memory^10,11,15–17^.

Together, this data descriptor and the publicly available code and data demonstrate the utility of NWB:N as an instrument to store, retrieve, and share cell-based electrophysiology data together with all associated meta data, stimulus information and behavior. This release additionally provides tools in both MATLAB and Python that will facilitate the adoption of NWB:N in the community of human intracranial recordings. Lastly, the experimental results shown confirm the reproducibility of previous results on the selectivity of MTL cells during the new/old task at a new study site, together with 17 new subjects that were not previously released.

## Methods

Although described extensively elsewhere^10^, here we briefly summarize details of the dataset, followed by NWB:N-specific methods which are specific to this data descriptor.

### Subjects

In total, we recorded from patients across 89 sessions (see Online-only Table 1) during intracranial monitoring of seizure activity in the epilepsy monitoring unit (EMU). Patients were admitted to the EMU to localize their seizure focus for potential surgical excision. Each patient has a recording-site specific identifier (H = Huntington Memorial Hospital, C = Cedars-Sinai Medical Center, T = Toronto Western Hospital). The number of sessions that an individual patient performed was variable. If the patient performed more than one session, a different variant of the task (with new images) was administered, thus allowing the patient to perform various versions of the task (either 1, 2, or 3 with different stimuli). All patients provided written informed consent to participate in the study. All protocols were approved by the Institutional Review Boards of the California Institute of Technology, the Huntington Memorial Hospital, Cedars-Sinai Medical Center, and Toronto Western Hospital.

### Task

The task consists of two parts: an encoding and a recognition phase^10^. In the encoding phase, subjects were presented with 100 novel images chosen from distinct visual categories (houses, landscapes, mobility, phones, animals, fruits, kids, military, space, cars, food, people, and spatial). Subsequently, in the recognition phase, subjects were presented with 50 “novel” images and 50 “old” images. During the recognition phase, subjects indicated whether they thought that the image was “novel” (never seen before), or “old” (seen during encoding) together with confidence ratings on a 1–6 scale. During the encoding phase, subjects indicated for each image whether it contained an animal or not (yes or no).

### Data acquisition

To isolate the activity of single neurons in the human MTL, we utilized hybrid depth electrodes with eight embedded microwires each (Ad-Tech Medical) as described previously^12^. The signal from each microelectrode was locally referenced to one of the eight microelectrodes. The continuously acquired raw signal was recorded with a Neuralynx ATLAS or Neuralynx Cheetah System (Neuralynx Inc.). Signals were recorded broadband (0.1 to 9000 Hz) and sampled at 32 kHz. Offline, each channel (i.e., microelectrode) was band-passed filtered from 300–3000 Hz before spike sorting.

Spikes were detected using threshold crossings of the local energy, or power, of the filtered signal, and sorted offline with the semiautomatic template-matching algorithm Osort^18^. To classify the detected clusters as putative units, we assessed the following criteria: (1) shape of mean waveform, (2) interspike interval distribution, (3) violation of the refractory period (<3% of the spikes have an ISI of less than 3 ms), and (4) stable firing rate and waveform amplitude during the task. For each isolated cluster, we computed several quality metrics for further analysis and quantification of spike sorting quality (isolation distance, mean waveform, and signal-to-noise ratio).

## Data Records

### NWB:N workflow: export

**Fig. 1:**
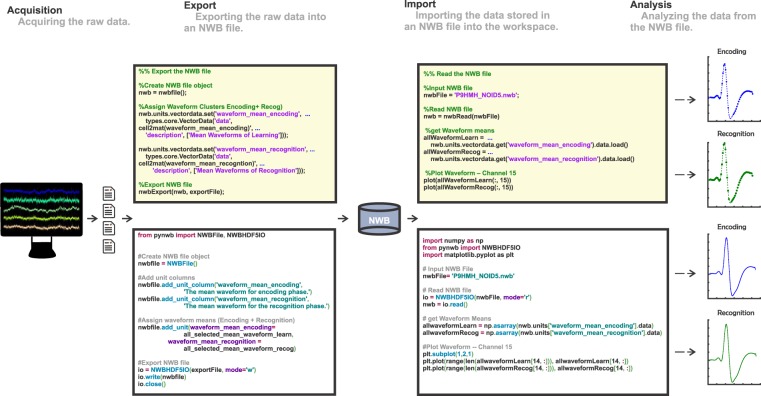
Overview of NWB workflow. Data is first acquired and stored in equipment/laboratory specific formats (left). This data is then read into MATLAB (top row) or Python (bottom row) and exported into NWB (middle). Subsequently, either MATLAB or Python can be used to read the NWB files and analyze the data (right). The example data loaded and plotted is the mean waveform of an individual neuron separately for the two phases of the task.

Our goal is to create NWB:N files that include all data used and acquired during the experiment as well as accompanying meta data that is needed for subsequent analysis (Fig. 1). In our case, the source data (stored in proprietary formats) that is exported includes: the stimuli (pictures) shown to subjects, behavioral responses (choices, reaction times), NEV (Neuralynx Event) files that indicate event markers (TTLs), spike times and waveforms from the OSort spike sorting software (‘Ax_cells.mat’ files, where x is the channel number), and information from the raw CSC (Continuously Sampled Channel) Neuralynx files. A variety of customized code is needed to read these files from their original data format. We use these tools to import the data either into MATLAB or Python and then utilize the NWB:N APIs to re-export the data for storage inside an NWB:N file (Fig. 1, left). This yields a single NWB:N file for each recorded session of the experiment. All data in both NWB and the native format have been deposited online^19^.

### Structure of the NWB file

**Fig. 2:**
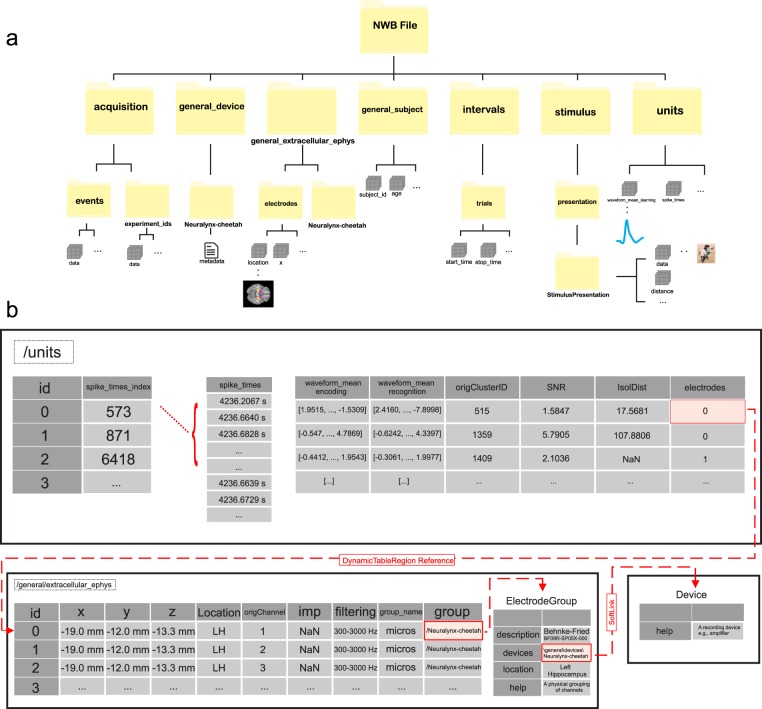
Organization of an NWB file when used for storing human single-neuron data. (**a**) Top-level structure of an NWB file. The top-level groups are acquisition, general, intervals, stimulus, and units. (**b**) Illustration of the \units (top) and \electrode (bottom) table. Shown are three example units (top) and three example electrodes (bottom). Notice how the electrode table refers to the Device table (right).

At the top-most level, an NWB file consists of several main groups, each of which are a container (similar to a directory) for different subsets of the data (see Fig. 2a for a summary). The main groups of interest here are acquisition (recorded raw data streams), intervals (epochs/trials), stimulus (stimulus data), units (spike times of isolated neurons), and general (metadata on devices, electrodes, and subject). Within each main group, different sets of pre-defined variables are part of the NWB:N specification. Each variable in NWB:N is of a pre-specified type, called ‘neurodata_types’. For each pre-specified type, a certain set of variables are mandatory, assuring standard compliance. For example, each Group is of type NWBContainer. Similarly, each Dataset specification within each Group is represented by the type NWBData, which all other base types, including Image, VectorData, DynamicTableRegion, and Index, inherit. Below, we next describe the elements that we utilized within the different top-level Groups (Fig. 2a).

A key goal of the NWB:N standard is to include all meta-data of each experiment within each NWB file. To achieve this, we have utilized the various meta-data fields within the NWB:N file to specify all the pertinent information needed to understand and analyze an experiment. Note that, in particular, many of the pre-specified data fields within the NWB file have a free text ‘description’ field that we utilized to add additional information. There are both structured/required meta-data fields such as the start time of the experiment (e.g., ‘session_start_time’), and descriptive/unstructured free text explanatory fields such as ‘description’ (a field that is part of many of the NWB data types used). Note that in order to protect PHI (patient health information), we had to omit or modify a small subset of the metadata provided. For instance, in the field session_start_time, we set only the year and month of the experiment but defaulted the actual day of the experiment to the first of the month for all sessions.

### NWB file content: acquisition group

The \acquisition Group contains the raw data and meta-data collected for each session that is essential to align the behavioral markers with the processed data. Two streams are included: \acquisition\events (‘events’) and \acquisition\experiment_ids (‘experiment_ids’). Both streams include the same number of entries in the same order.

\Events stores data and timestamps along with a meta-data field (‘description’) that details the meaning of the behavior markers. Data stores the event markers (i.e., TTLs) of the experiment (see Table 1 for a summary). The following TTL values are used: Start of Experiment (55), Stimulus Onset (1), Stimulus Offset (2), Question Screen Onset (3), New/Old Response (20 or 21), Confidence of Response (31–36), End of Trial (6), End of Experiment (66). For the learning block, at the time marked as “Question Screen Onset” (TTL = 3), the question “Is this an animal?” is shown. There are two possible answers, which are encoded as either 20 (Yes, this is an animal) or 21 (No, this is not an animal). For the recognition block, at the time marked as “Question Screen Onset” (TTL = 3), the question “Have you seen this image before?” is shown. There are six possible answers, which are encoded as TTLs 31–36 [31 (new, confident), 32 (new, probably), 33 (new, guess), 34 (old, guess), 35 (old, probably), 36 (old, confident)]. The timestamps (recorded in seconds relative to start of the experiment) record the time each experiment marker occurred.

**Table 1:** Event markers (“TTLs”) used.

Event ID	Description
55	Start of Experiment
1	Stimulus Onset
2	Stimulus Offset
3	Question Screen Onset
20, 21	Response During Learning^a^
31–36	Response During Recognition^b^
6	End of Trial
66	End of Experiment

^a^During the learning phase, subjects are instructed to respond to the following question: “Is this an animal?” in each trial. Response are encoded as “Yes, this is an animal” (20) and “No, this is not an animal” (21). ^b^During the recognition phase, subjects are instructed to respond to the following question: “Have you seen this image before?” in each trial. Responses are encoded as: 31 (new, confident), 32 (new, probably), 33 (new, guess), 34 (old, guess), 35 (old, probably), 36 (old, confident). The ‘description’ field within \acquisition\events of the NWB file also contains the information listed in this table.

For every entry in \Events, there is also an entry in \experiment_ids that stores the following attributes: data and timestamps. Here, data refers to the trial type, either learning or recognition with the corresponding timestamps (events and experiment_ids has the same number of entries, thereby assigning each TTL to an experiment). This information is used to designate which block a trial corresponds to. The learning block is labeled with only one of the following: 80, 83, or 88, while the recognition block is labeled with only one of the following: 81, 84, or 89 (see Table 2 for a summary). The experiment_ids vary only so that different runs of the same experiment can be disambiguated.

**Table 2:** Experiment IDs used.

Experiment ID	Description
80, 83, 88	Learning Phase
81, 84, 89	Recognition Phase

The learning and recognition phase are denoted by the IDs listed. A session will have one of the following ID pairs (learning, recognition): (80, 81), (83,84) or (88,89). The ‘description’ field within \acquisition\experiment_ids of the NWB file indicates the experiment ID used for each phase of the experiment of that particular session.

### NWB file content: general group

Second, the \general Group contains metadata about the experiment (Fig. 2a). There are several sub-groups: general\devices (‘devices’), general\extracellular_ephys (‘extracellular_ephys’), and general\subject (‘subject’). Devices documents the device(s) used for signal acquisition, which here is the Neuralynx Inc. amplifier (“Neuralynx-Atlas”) or (“Neuralynx-cheetah”). Other signal acquisition systems can be indicated here accordingly by adding a new entry to ‘devices’. General/extracellular_ephys contains information about the electrodes recorded from, including their location (brain area and coordinates), impedance, and filters used (Fig. 2b, bottom). This information is combined in the electrodes table, which is part of the extracellular_ephys group. For example (see Fig. 2b), the \electrodes table identifies that ‘neuron1’ has id 0, was recorded in the Left Hippocampus (*location*) with (−19.0 mm, −12.2 mm, −13.3 mm) as the MNI coordinates (*x, y, z*), and the filter applied before spike sorting was 300–3000 Hz. The origChannel (a custom column) refers to the hardware channel that was used to record from this electrode. An explicit object reference in the ‘group’ column of the \electrodes table links to an ElectrodeGroup, which contains additional information about the electrodes used. Here, the information provided is that the electrodes were microwires. The ‘device’ soft link (Fig. 2b, lower right) within the ElectrodeGroup contains an object reference to the Device group (/general/devices), which provides additional metadata about the electrodes and recording system used (here, we used one entry to describe the combination of both). Lastly, the general/subject group contains meta-data about the subject (age, description, sex, species, and subject id).

### NWB file content: interval group

The \intervals Group contains information about individual trials in the field \trials. It contains the following trial attributes: start_time, stop_time, delay1_time, response_time, delay2_time, new_old_labels_recog, response_value, category_name, stimCategory, and stim_phase. There is one entry for every trial. Start_time is the time of stimulus onset of each trial, and stop_time is the time of stimulus offset. Delay1_time is the time of the question screen onset, and response_time records the time the subject provided a response. Delay2_time indicates the end of the trial. All times are in seconds. The remaining attributes provide additional information about each trial: response_value is the response (button press) given by the subject to the stimuli shown (see acquisition group for details on the response values), while response_time indicates the time of the response relative to the start of the experiment, stim_phase describes the part of the experiment this trial belongs to (learning or recognition), category_name and stimCategory indicates the visual category the image shown belongs to (as a string and number, respectively). New_old_labels_recog provides the ground truth label of whether the trial showed a new or old stimulus during the recognition phase (0 is old, 1 is new).

### NWB file content: stimuli group

The \stimuli Group stores the stimuli (i.e., images) presented during the experiment. Each stimulus is listed within stimuli\presentation\ as stimuli_learn_x and stimuli_recog_x, with x = 1…100. The actual image is stored within each as the data attribute. There are a total of 200 trials (100 encoding trials and 100 recognition trials). The order corresponds to the order of stimuli presented during the task with the category of each stimulus specified within \intervals\category_name.

### NWB file content: units group

The \units Group contains information about all recorded units (“single neurons”) after spike sorting, including their electrophysiological features (e.g., spikes, waveforms, etc.). The \units table is a column-based DynamicTable, where each column enumerates a different feature (Fig. 2b, top). The \units table permits the storage of a variable number of columns, including required as well as optional and custom columns. We use the following columns: spike_times_index, spike_times, waveform_mean_encoding, waveform_mean_recognition, origClusterID, SNR, IsolDist, and electrodes. Each row denotes a different isolated neuron (indexed by id), with one entry per row for each column. The column spike_times_index is a link into the ragged array spike_times, which contains a concatenation of all spike times of all neurons (and thus has many more rows than neurons). The spike_times_index column value refers to the last spike of each neuron, thus indicating the range of spikes that belong to this neuron starting at +1 relative to the last neuron till the value provided. The two waveform_mean columns contain the extracellular waveform of the neuron for the two task phases (sampled at 100 kHz). The electrodes column provides a link to a range of entries in the \electrodes table, thereby providing the electrode(s) this neuron was recorded from (stored in the \electrodes table). This is done with a DynamicTableRegion reference to accommodate tetrodes and other types of electrodes, but here this link is 1:1 since each neuron is only visible on one channel. Lastly, SNR and IsolDist provide spike quality metrics and origClusterID provides the original cluster ID generated by the spike sorting algorithm (here, OSort; thereby providing a link to the original data).

### Implementation of export to NWB of events data

We use the standard NWB:N APIs to export the data stored in custom-format Matlab files to NWB:N as described above. To illustrate a specific case of how this is achieved we here describe in detail an example showing how to export the acquisition top-level group (which contains a list of TTLs) using both MATLAB and Python. The code snippets below illustrate how to achieve this in MATLAB with matNWB and in Python with PyNWB, respectively.

MATLAB:

1. nwb = nwbfile()
2. load(‘eventsRaw.mat’)
3. nwbEvents = types.core.AnnotationSeries(‘data’, num2str(events(:, 2)), ‘data_unit’, ‘NA’, ‘timestamps’, events(:, 1), ‘description’, ‘TTLs’)
4. nwb.acquisition.set(‘events’, nwbEvents)

Python:

1. nwb = NWBFile(…)
2. events = loadmat(‘eventsRaw.mat’)[‘events’]
3. nwbEvents = AnnotationSeries(name = ‘events’, unit = ‘NA’, data = np.asarray(events[:, 1]), timestamps = np.asarray(events[:, 2]), description = ‘Export events to NWB file’)
4. nwb.add_acquisition(nwbEvents)

This example demonstrates that apart from syntax differences, usage of the APIs is similar in both languages. First, calling nwbfile() instantiates a new NWB object (Line 1). Then, a AnnotationSeries is created (Line 3), which is then added to the \acquisition Group (Line 4). Both snippets shown above produce identical nwb files, thereby providing interoperability. See the additional code files demoExportEvents.m & demoExportEvents.py for the full version.

### Implementation of export to NWB of units data

As a second use case, we next demonstrate how to populate aspects of the units table, which is a DynamicTable. In MATLAB, the steps to assign the spike_times and spike_times_index columns are:

1. nwb = nwbfile()
2. [spike_times_allCells] = getSpikeTimesAllCells(…)
3. nwb.units = types.core.Units(‘colnames’, ‘spike_times’, ‘description’, ‘Units Table’)
4. [spike_times_vector, spike_times_index] = create_indexed_column(spike_times_allCells, ‘/units/spike_times’);
5. nwb.units.spike_times = spike_times_vector;
6. nwb.units.spike_times_index = spike_times_index;
7. nwb.units.id = types.core.ElementIdentifiers(‘data’, int64([1:length(spike_times_index.data)]-1));

Similarly, to achieve the same in Python, the steps are^2^:

1. nwb = NWBFile(…)
2. allSpikeTimes = demoHelper.getSpikeTimesAllCells(…)
3. for cluster in allSpikeTimes.keys():
4. nwb.add_unit(id = int(cluster), spike_times = allSpikeTimes[cluster][0])

See the additional code files demoExportSpikeTimes.m & demoExportSpikeTimes.py for the full version.

### Implementation of import of NWB data

Both matNWB and PyNWB also provide an API for reading existing NWB files. However, the ways of how to access and parse the resulting data differs somewhat between the two. For instance, the steps to query the ‘waveform_mean_encoding’ column in the units table (which is a user-created column) via matNWB are as follows:

1. nwb = nwbRead(‘P9HMH_NOID5.nwb’)
2. waveform_mean = nwb.units.vectordata.get (‘waveform_mean_encoding’).data.load()

In comparison, the steps to query the same data via PyNWB are:

1. io = NWBHDF5IO(‘P9HMH_NOID5.nwb’, mode = ‘r’)
2. nwb = io.read()
3. waveform_mean = np.array(nwb.units[‘waveform_mean_encoding’].data)
4. io.close()

Apart from syntactical differences, the overall protocol for reading the NWB-formatted data remain the same across both languages. First, the NWB file is read using the NWB read utility; subsequently, the ‘waveform_mean_encoding’ is extracted from the \units DynamicTable, where each row of waveform_mean_encoding represents the mean waveform for an individual neuron.

As a second example, we next illustrate how to read the spike_times from the \units table. The steps to retrieve all the ‘spike_times’ of a specific neuron (here, neuron nr. 3) with matNWB are:

1. nwb = nwbRead(‘P9HMH_NOID5.nwb’)
2. allSpikes = nwb.units.spike_times.data.load()
3. spike_times_index = nwb.units.spike_times_index.data.load()
4. neuron3 = allSpikes(spike_times_index(2) +1:spike_times_index(3))

The steps to achieve the same with PyNWB are as follows:

1. with NWBHDF5IO(‘P9HMH_NOID5.nwb’, mode = ‘r’) as io:
2. nwb = io.read()
3. neuron3 = np.array(nwb.units.get_unit_spike_times(2))

## Technical Validation

**Fig. 3:**
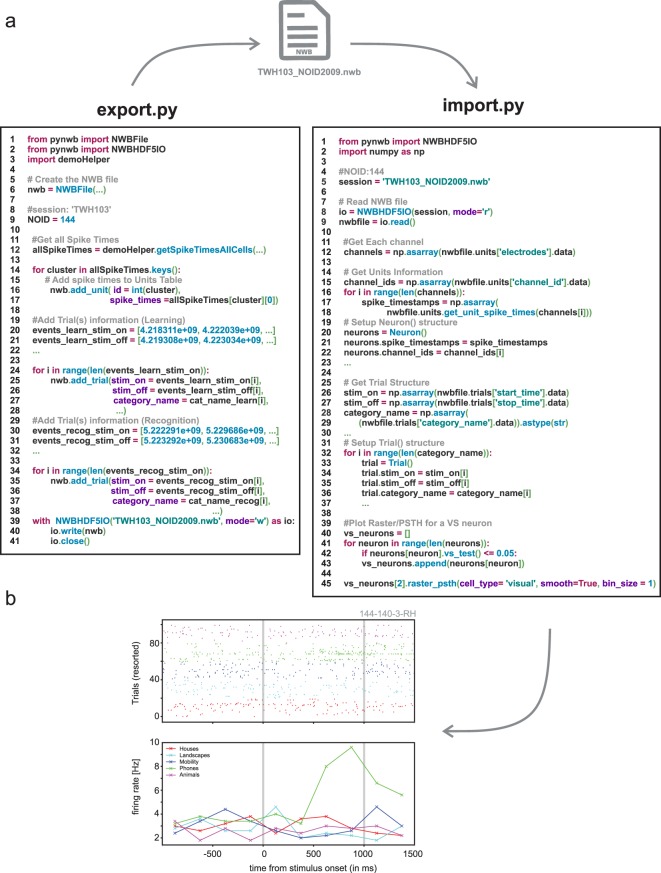
Illustration of analysis pipeline in Python. Shown is how spike times and trial information of the native dataset is exported into NWB (left) and how this data is subsequently read from NWB for plotting (right). An example VS neuron selective for the visual category of phones is shown at the bottom (The ID of this neuron 144-140-3-RH corresponds to: session ID – channel number – cell number – brain area).

**Fig. 4:**
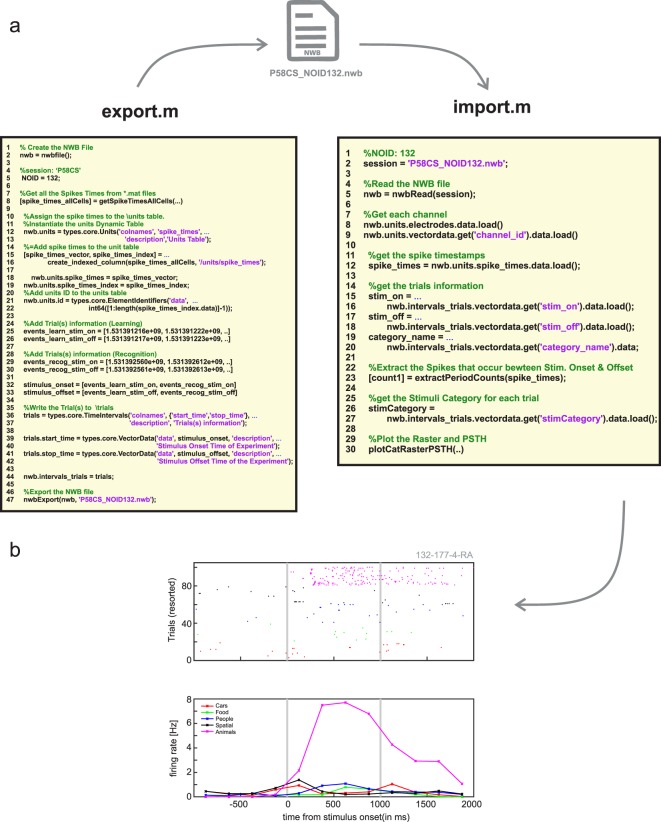
Illustration of analysis pipeline in MATLAB. Shown is how spike times and trial information of the native dataset is exported into NWB (left) and how this data is subsequently read from NWB for plotting (right). An example VS neuron selective for the visual category of animals is shown at the bottom (The ID of this neuron 132-177-4-RA corresponds to: session ID – channel number – cell number – brain area).

To demonstrate the utility of NWB:N as a platform to store and analyze human single-neuron data, we developed easy-to-use pipelines of code to export existing data into NWB:N and to read back the resulting NWB files for analysis. To highlight the interoperability of NWB:N, we developed the identical pipeline in both Python and MATLAB (Figs. 3 and 4). As a first step, we begin by showing how data recorded from a single neuron is exported to NWB:N and then re-imported and plotted. We then describe the full pipeline, followed by key results that reproduce previous experimental results we published for this dataset.

### Simple use case: importing and exporting a single neuron to/from NWB

Export.py highlights the process of exporting the spike times and trial(s) information of a single neuron with Python (Fig. 3, left). Information about the trials (e.g., stimulus onset, stimulus offset, etc.) is added to the trials group (\intervals\trials) by calling add_trial(…), where the arguments are the individual trial attributes, resulting in an array of trial times. The point of time at which each neuron fired spikes (spike times) are added to the units table (\units) via the add_unit(…) method, resulting in a concatenated set of spike times. Lastly, the file is exported by utilizing NWBHD5IO.

Import.py highlights the process of importing data from an NWB file for plotting (Fig. 3, middle). To retrieve all spike_times of a channel (which could be multiple neurons), get_unit_spike_times(…) is called, followed by instantiation of the Neuron() class. Trial information is retrieved from \intervals\trials by directly indexing the different columns (start_time, stop_time, category_name), followed by instantiation of the Trial() class. Note that the Neuron and Trial classes are custom and not part of the NWB:N API. The data is now ready for analysis in plotting, which here is performed by the raster_psth(…) method, which produces the raster plot and Peri-Stimulus Time Histogram (PSTH) shown in Fig. 3 (right).

Export.m and import.m illustrate the same process, but for MATLAB using the matNWB API (Fig. 4). The usage of the two APIs differs in important ways, so we here detail the differences. First, instead of the add_unit helper function, an instance of types.core.Units(..) is constructed, followed by manual insertion of the index column spike_times and spike_times_index using create_indexed_column. Similarly, instead of add_trial, types.core.TimeIntervals(…) is instantiated and populated manually before assigning it to the \intervals_trials group. For importing data, the data is accessed directly using nwb.units.spikes_times.data.load(…) and nwb.intervals_trials.vectordata.get(…). The method plotCatRaster(…) shows how to use the imported data for plotting the example raster and PSTH shown in Fig. 4 (right).

### Full processing pipeline

In this section, we briefly outline the full processing pipeline that is part of this release. These functions serve as easy to adapt templates for usage with other human single-neuron datasets. In the next section, we then proceed to summarize the key analysis results that this processing pipeline produces (summarizing across the entire dataset).

**Fig. 5:**
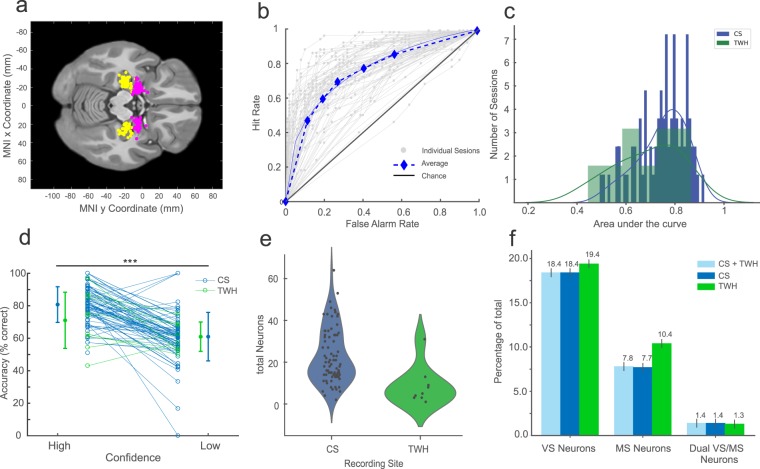
Summary of experimental result computed using the NWB-based processing pipeline. (**a**) Summary of recording locations. Each dot is a different electrode. Shown is an Axial View (z = 81) of the CTI168 MRI atlas^22^ (Pink: Amygdala, Yellow: Hippocampus). (**b**) Behavioral ROC curves for all individual sessions (grey) and the average across all sessions (Blue). Each dot is a different confidence level, with the highest confidence (6) equal to the dot with the lowest false alarm rate. (**c**) AUC values for all CS (Green) and TWH sessions (Blue). The average AUC was 0.74 ± 0.09 and 0.69 ± 0.13, respectively. (**d**) Accuracy was significantly different for high vs. low confidence trials (***P < 0.001, paired *t* test) for all sessions. Each line shows an individual session with the standard deviation for CS sessions (Blue) and TWH sessions (Green). (**e**) Total number of neurons recorded from the MTL in each session at Cedars-Sinai Medical Center (CS) and Toronto Western Hospital (TWH). Each dot is a session. (**f**) Proportion of VS, MS, and Dual VS/MS cells selected from the entire dataset (CS + TWH) as well as separately for the sessions acquired at CS and TWH. Note that throughout this figure, “CS” refers to all sessions with labels starting with either C or H, whereas TWH refers to all session with labels starting with T (see Online-only Table 1, column 1).

In python, the main export routine is *no2nwb_main.py*, which lists the parameters needed to export the native data into NWB:N. All the sessions with corresponding metadata are enumerated in the configuration file (defineNOsessions_release.ini). The main function called from within *no2nwb_main.py* is *no2nwb.py*, which contains the central NWB:N methods to export the data. To read from and organize the native data, *data.py* defines the NOData class, which facilitates the export of the native data into an organized structure. Specifically, NOData imports the Cell class (defined in *cell.py*) and the Trial class (defined in *trial.py*) that enable this organization. The main analysis routine is *main.py*, which partitions the analysis into behavior and single neuron. Main.py reads in only NWB:N files, thereby confining the analysis to the components of the NWB:N file. The behavioral analysis is implemented in behavior.py and behavioral_all.py (see Fig. 5). The single neuron analysis is implemented in single_neuron.py, which computes the raster plot and PSTH (see Figs. 3 and 4).

In MATLAB, the main export routine is *exportNO2NWB_main.m*, which contains the pertinent methods of exporting the native data into NWB:N. All the parameters needed to run this script are detailed at the beginning. *ExportNO2NWB.m* reads in the native data, structures it, and then writes the data into an NWB:N file. *NWBexport_accumulateCells.m* helps store components of the native data before exporting it into an NWB file. The main analysis routine is *NWBneural_main.m*, which, like the python analysis routine noted above, reads in only NWB:N files. Within *NWBneural_main.m*, behavioral analysis is defined by *NWB_behaviorSummary.m*, while the single neuron analysis is defined by *NWBneural_loopOverSessions_release.m*. For the behavior, *NWBloadDataOfBlock_release.m* reads in the NWB file for each specified session, while *NWBrunForAllCellsInSession.m* reads in the NWB file for the single neuron analysis.

### Key experimental results: behavior

Subjects performed a recognition memory task with two distinct phases, an encoding phase followed by a recognition phase (see methods)^10,11^. To quantify the quality of the subject’s memory, we performed a Receiver Operating Characteristic (ROC) analysis. We used the area under the curve (AUC) of the ROC to quantify the ability of subjects to successfully differentiate between new and old stimuli (Fig. 5b). The average AUC across all sessions was 0.74 ± 0.10 (Fig. 5c; performance was similar across the two study sites, with an average AUC for CS and TWH sessions of 0.74 ± 0.09 and 0.69 ± 0.13, respectively). Also, the shape of the ROC curve was asymmetric (Fig. 5b, p < 0.05), as expected for declarative memories^20^. We next assessed whether subjects were able to judge the quality of their memories by comparing accuracy separately for trials in which subjects indicated high vs. low confidence. Accuracy was significantly larger in high compared to low confidence trials (P < 0.001) (Fig. 5d) across all sessions. These behavioral results demonstrate that subjects utilized declarative memories to make subjective memory-based decisions about the novelty and familiarity of images^21^.

### Key experimental results: proportion of selective cells

We previously reported on the details of two kinds of cells with different response profiles in this dataset: Visually Selective (VS) and Memory Selective (MS) cells^10,11^. Here, we repeated a few of these key analyses to demonstrate that the proportions of these cells are as expected in this dataset (particularly in the not previously analyzed part of the data). The second goal of repeating this analysis is to illustrate how to perform the analysis steps with the NWB-formatted data.

The response of VS cells is tuned to the visual category of the stimulus, responding preferentially to images of a particular visual category during the stimulus presentation window (see Figs. 3 and 4 for an example). To select for VS cells, we performed a 1 × 5 ANOVA (one way, P < 0.05) to test whether the firing rate during the retrieval trials in a 1 s long window starting 200 ms after stimulus onset was related to the visual category (there are 5 different categories in each experiment). The response of MS cells differentiates between novelty and familiar stimuli, responding preferentially to either new (Novelty Selective) or old (Familiarity Selective) stimuli. To select for an MS cell, we compared the firing rate between the 50 novel and 50 familiar trials during the retrieval period in a 1 s window starting 200 ms after stimulus onset (two-tailed bootstrap comparison of means with 1,000 runs, P < 0.05^11^).

We recorded a total of 1863 neurons in the MTL (Fig. 5a). Out of these 1863 cells (Fig. 5e), 343 cells qualified as VS cells (18.4%) and 146 cells qualified as MS cells (7.8%). 26 cells qualified as both VS and MS cells (1.4%) (Fig. 5f). Compared to previously published results^10,11^, this dataset includes recordings from 23 new sessions, including 9 sessions from a new institution (Toronto Western Hospital, TWH) at which we recorded this experiment as part of the NIH Brain initiate (Fig. 5e). To demonstrate reproducibility of the key results in the NWB format, we next compared the proportion of isolated functional cell types between the existing and new dataset. We found that the percentages of VS, MS, and dual VS/MS cells were comparable to previously reported percentages in our locally recorded dataset (Fig. 5f)^10^. Together, this analysis shows that the basic results reproduce in this newly acquired dataset and that the results from the NWB:N-based pipeline are as expected based on previous work using a different processing pipeline on this same dataset.

## Data Availability

All code associated with this project is available as open source. The code is available on GitHub under the BSD license (https://github.com/rutishauserlab/recogmem-release-NWB). Both Python and MATLAB scripts are included in this repository along with the matNWB API. We also provide a streamlined workflow as a Jupyter Notebook. Note, we tested our code with the following versions of the Python Packages: numpy (1.17.2), pandas (0.23.0), scipy (1.1.0), matplotlib (2.2.2), pynwb (1.1.0), hdmf (1.2.0), and seaborn (0.9.0). Detailed instructions on installing and running the code in this repository are found in our online documentation on GitHub.

## Online-only Table

**Online-only Table 1:** List of all Patients.

ID	Number of Sessions	Session ID(s)	Variant(s)	Age	Sex	Epilepsy Diagnosis (based on intracranial recordings)
H09	2	5, 6	1, 2	55	M	Right Mesial Temporal
H10	1	7	1	37	M	Left Frontal
H11	1	9	1	16	M	Right Lateral Frontal
H14	2	17,18	2, 1	31	M	Bilateral Independent Temporal
H15	2	20, 21	1, 2	45	M	Right Mesial Temporal
H16	2	23, 24	2, 1	34	F	Right Frontal
H17	1	32	1	19	M	Left Inferior Frontal
H18	1	26	1	40	M	Right Mesial Temporal
H19	2	27, 28	2, 1	34	M	Left Frontal
H21	3	38, 39, 41	2,1,3	20	M	Not Localized
H23	3	43, 44, 47	3, 2, 1	40	M	Left Mesial Temporal
H27	1	58	2	40	M	Bilateral Independent Temporal
H28	1	48	3	22	M	Right Mesial Temporal
H29	1	49	3	17	F	Left Deep Insula
H31	1	50	2	30	M	Right Mesial Temporal
H33	1	52	1	29	M	Left Mesial Temporal
H42	2	54, 55	1, 1	29	M	Not Localized
H43	1	56	1	27	F	Left Mesial Temporal
H44	1	68	1	57	F	Right Mesial Temporal
H47	2	92, 97	1, 3	20	M	Right Mesial Temporal
H48	1	99	1	54	M	Left Mesial Temporal
H51	2	104, 105	3, 1	24	M	Bilateral Frontal and Temporal
C24	2	59, 60	1, 2	47	F	Not Localized
C25	2	61, 63	1, 2	36	F	Bilateral Independent Mesial Temporal
C26	2	64, 66	1, 2	56	F	Left Mesial Temporal
C27	1	67	1	44	M	Left Mesial Temporal
C29	2	69, 70	1, 2	19	M	Left Neocortical Temporal
C31	1	73	3	32	M	Left Neocortical Temporal
C32	1	74	2	19	M	Not Localized (Generalized)
C33	3	76, 77, 78	3, 2, 1	44	F	Right Mesial Temporal
C34	1	85	3	70	M	Bilateral Mesial Temporal
C37	1	96	3	33	F	Right Mesial Temporal
C38	1	102	3	63	F	Right Mesial Temporal
C39	2	93, 100	1, 3	26	M	Right Insula
C40	1	101	3	25	M	Right Motor Cortex
C42	2	111, 112	1, 2	25	F	Not Localized
C43	1	113	3	42	F	Left Mesial Temporal
C44	1	114	3	53	F	Right Mesial Temporal
C47	1	115	3	32	M	Right Mesial Temporal
C48	1	116	3	32	F	Left Mesial Temporal
C49	2	117, 118	3, 1	24	F	Left Mesial Temporal
C51	2	119, 120	3, 1	17	M	Not Localized (No Seizures)
C53	2	122, 123	3, 1	60	M	Bilateral Independent Mesial Temporal Lobe
C54	3	124, 125, 126	3, 1, 2	59	F	Right Mesial Temporal
C55	1	127	1	43	F	Right Mesial Temporal
C56	1	128	2	48	M	Multifocal, Bitemporal + Orbitofrontal
C57	2	130, 131	3, 2	46	M	Left Neocortical Parietal
C58	2	132, 133	3, 1	32	F	Right Frontal Neocortical
C60	1	134	1	67	M	Left Mesial Temporal
C61	1	135	1	52	F	Left Mesial Temporal
C62	1	136	1	25	F	Right Mesial Temporal
T88	2	2001, 2002	2, 3	26	M	Right Mesial Temporal
T89	1	2004	1	45	F	Right Frontal
T90	1	2003	3	20	M	Occipital Cortex
T98	1	2005	1	46	F	Left Neocortical Temporal
T100	1	2008	1	19	F	Right Insula
T101	1	2011	1	25	F	Left Neocortical Temporal
T103	1	2009	1	49	M	Right Mesial Temporal
T107	1	2007	1	64	F	Left Mesial Temporal
Total Subjects: 59	**Total Sessions: 87**		**Total Female: 25**		**Total Male: 34**	

Each patient has an identifier (ID), denoting the Institution and the corresponding patient number (H = Huntington Memorial Hospital, C = Cedars-Sinai Medical Center, T = Toronto Western Hospital). Each session has a unique session ID (also referred to as “NOID”). Sessions are referred to by their session ID both in defineNOsessions_release.ini (native data) and the NWB formatted file within the variable\session_description.
